# Intratumoral and peritumoral radiomics model based on abdominal ultrasound for predicting Ki-67 expression in patients with hepatocellular cancer

**DOI:** 10.3389/fonc.2023.1209111

**Published:** 2023-08-24

**Authors:** Hongwei Qian, Zhihong Shen, Difan Zhou, Yanhua Huang

**Affiliations:** ^1^ Department of Hepatobiliary and Pancreatic Surgery, Shaoxing People’s Hospital, Shaoxing, China; ^2^ Shaoxing Key Laboratory of Minimally Invasive Abdominal Surgery and Precise Treatment of Tumor, Shaoxing, China; ^3^ Department of Ultrasound, Shaoxing People’s Hospital, Shaoxing, China

**Keywords:** hepatocellular cancer, ultrasonography, Ki-67 Antigen, radiomics, machine learning, computer assisted diagnosis

## Abstract

**Background:**

Hepatocellular cancer (HCC) is one of the most common tumors worldwide, and Ki-67 is highly important in the assessment of HCC. Our study aimed to evaluate the value of ultrasound radiomics based on intratumoral and peritumoral tissues in predicting Ki-67 expression levels in patients with HCC.

**Methods:**

We conducted a retrospective analysis of ultrasonic and clinical data from 118 patients diagnosed with HCC through histopathological examination of surgical specimens in our hospital between September 2019 and January 2023. Radiomics features were extracted from ultrasound images of both intratumoral and peritumoral regions. To select the optimal features, we utilized the t-test and the least absolute shrinkage and selection operator (LASSO). We compared the area under the curve (AUC) values to determine the most effective modeling method. Subsequently, we developed four models: the intratumoral model, the peritumoral model, combined model #1, and combined model #2.

**Results:**

Of the 118 patients, 64 were confirmed to have high Ki-67 expression while 54 were confirmed to have low Ki-67 expression. The AUC of the intratumoral model was 0.796 (0.649-0.942), and the AUC of the peritumoral model was 0.772 (0.619-0.926). Furthermore, combined model#1 yielded an AUC of 0.870 (0.751-0.989), and the AUC of combined model#2 was 0.762 (0.605-0.918). Among these models, combined model#1 showed the best performance in terms of AUC, accuracy, F1-score, and decision curve analysis (DCA).

**Conclusion:**

We presented an ultrasound radiomics model that utilizes both intratumoral and peritumoral tissue information to accurately predict Ki-67 expression in HCC patients. We believe that incorporating both regions in a proper manner can enhance the diagnostic performance of the prediction model. Nevertheless, it is not sufficient to include both regions in the region of interest (ROI) without careful consideration.

## 1 Introduction

Hepatocellular carcinoma (HCC) is one of the most commonly diagnosed cancer, with more than 700,000 new HCC cases and 600,000 deaths in the world every year (1). Although surgical resection is the primary treatment for HCC with well-preserved liver function, the 5-year survival rate is only 10%-20% (2), due to a high recurrence rate after operation (3).

Ki-67 is an antigen associated with cell proliferation (4), playing a role in the therapeutic response and prognosis of malignant tumors (5). The Ki-67 proliferation index (PI) is commonly used as a prognostic indicator in various cancers (6–8). In HCC patients, high Ki-67 expression is associated with aggressive tumor characteristics and adverse outcomes (9). Accurately identifying Ki-67 expression is crucial, and the current evaluation of Ki-67 mainly depends on surgical pathology or needle biopsy. However, surgical pathology is a time-consuming process, and needle biopsy may not capture the complete heterogeneity of the tumor. Therefore, a noninvasive preoperative approach is needed to predict Ki-67 status and guide personalized treatment in HCC patients.

Previous research has indicated that radiomics (10), which involves converting medical images into imaging features and selecting those highly related to tumors, holds the potential to predict tumor phenotype, classification, stage, and other biological behaviors (11–13). For instance, Wu et al. (14) analyzed computer tomography (CT) findings of HCC patients and predicted the Ki-67 expression level based on texture features extracted from CT images. Similarly, Fan et al. (15) developed a nomogram based on radiomics features and clinical factors from enhanced magnetic resonance imaging (MRI) images, showing promising diagnostic performance.

The peritumoral tissue, which is the tissue surrounding the tumor, can also provide valuable information about tumor initiation and progression (16). By using radiomics to analyze peritumoral tissue, it is possible to predict Ki-67 expression in tumors (17). And in HCC, peritumoral tissue was thought to be associated with MVI and invasiveness (18, 19).However, few studies have evaluated the relationship between peritumoral tissues and Ki-67 expression in HCC patients.

CT, MR, and US play crucial roles in the evaluation of HCC: CT can provide high-resolution images and has high sensitivity and specificity for diagnosing HCC. MR can provide more detailed anatomical and functional information, such as liver metabolism and perfusion. US is a non-invasive, real-time, and repeatable imaging technique that holds high value for early diagnosis and treatment monitoring of HCC (20, 21). By combining radiomics, ultrasound has the potential to offer enhanced insights into HCC (22). Against this backdrop, our study aimed to assess the predictive value of radiomics features extracted from intratumoral and peritumoral tissues using abdominal ultrasound in HCC patients for Ki-67 status. We aimed to develop and validate an abdominal ultrasound radiomics model, and to investigate the associations between radiomics and Ki-67 expression in HCC patients.

## 2 Materials and methods

### 2.1 Study population

**Figure 1:**
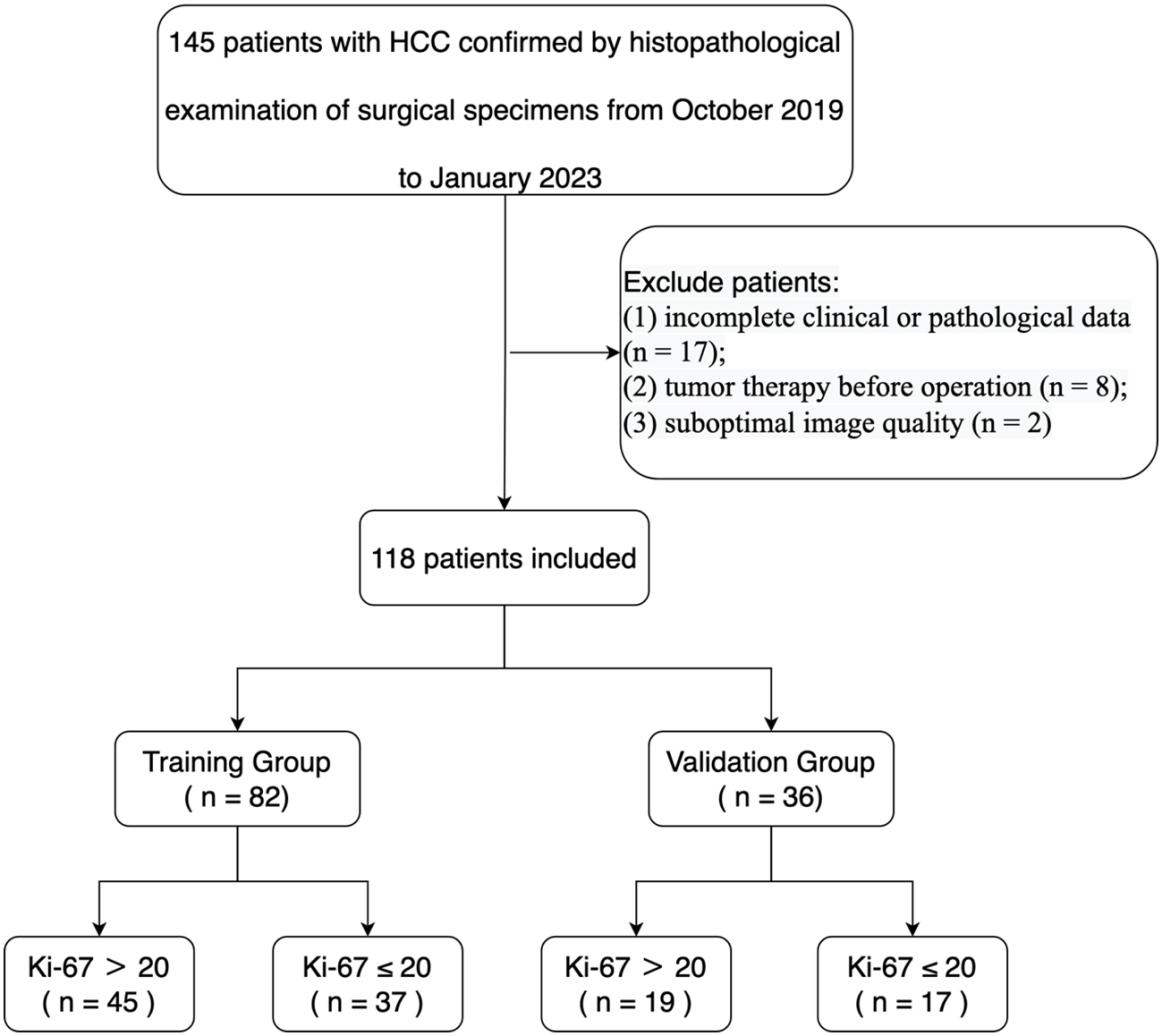
Flowchart of the inclusions and exclusions criteria of participants.

Our study received ethical approval from our institutional ethics committee. We conducted a retrospective analysis of ultrasonic and clinical data from 145 patients diagnosed with HCC by histopathological examination of surgical specimens in our hospital from September 2019 to January 2023. The assignment of the pathological diagnosis for each case was conducted in accordance with the 2019 WHO Classification of Tumors of the Digestive System (23). The inclusion criteria were as follows (1): ultrasound examination performed within 1 week before the operation (2); age of 18 or older (3); pathologically confirmed HCC (4); complete pathological data, including the Ki-67 proliferation index (5); for patients with multiple lesions, the largest lesion with matched pathological and immunohistochemical diagnosis was selected. Exclusion criteria were (1): incomplete clinical or pathological data (n = 17) (2); tumor therapy prior to the operation (n = 8) (3); suboptimal image quality (n = 2). Ultimately, 118 HCC patients were enrolled in the study, and they were randomly assigned to a training group (n = 82) and a testing group (n = 36) in a 7:3 ratio (**Table 1**). A flowchart illustrating the inclusion and exclusion of patients is presented in **Figure 1**.

**Table 1:** Comparison of clinical characteristics between the high Ki-67 expression group and low Ki-67 expression group.

Variables	High Ki-67 Group	Low Ki-67 Group	P
**Age(year)**	63.89 ± 10.96	66.43 ± 10.96	0.217
**Sex**			0.454
Male	49	43	
Female	16	10	
**HBsAg**			0.712
Positive	45	35	
Negative	20	18	
**AFP (mg/mL)**	2482.87 ± 11223.84	845.03 ± 4714.03	0.327
**Alb (g/L)**	38.61 ± 4.53	38.87 ± 4.09	0.746
**ALT(IU/L)**	38.24 ± 37.42	40.53 ± 36.98	0.743
**AST(IU/L)**	42.58 ± 46.88	46.16 ± 38.91	0.66
**TBIL (µmol/L)**	17.96 ± 20.1	16.2 ± 8.75	0.558
**DBIL (µmol/L)**	6.18 ± 11.42	6.12 ± 5.08	0.971
**PT (s)**	13.11 ± 1.42	13.17 ± 1.34	0.806
**INR**	1.05 ± 0.14	1.06 ± 0.11	0.664
**Tumor Size(cm)**	4.85 ± 2.77	5.0 ± 2.85	0.769
**Cirrhosis**			0.259
Absent	30	30	
Present	35	23	
**Multifocality**			0.371
Absent	51	45	
Present	14	8	

AFP, alpha fetoprotein; ALB, albumin level; ALT, alanine aminotransferase; AST, aspartate aminotransferase; TBIL, total bilirubin; DBIL, directed bilirubin; PT, prothrombin time; INR, international normalized ratio.

Multifocality absent means only one tumor lesion (51 cases in the High Ki-67 group and 45 cases in the Low Ki-67 group), multifocality present means there are multiple tumor lesions (14 cases in the High Ki-67 group and 8 in the Low Ki-67 group).

### 2.2 Image acquisition

The studies were done with patients lying in a supine position with both arms elevated above the head. And the image of the tumor at the largest diameter was saved in digital imaging and communications in medicine (DICOM) format for further investigation.

Ultrasound examination was performed by using one of the following ultrasound machines: LOGIQ E8 (GE Healthcare, United States; C5-1 convex array probes, 1–5 MHz); LOGIQ E9 (GE Healthcare, United States; C5-1 convex array probes, 1–5 MHz); Aplio 500 (Toshiba Medical systems, Japan; 6C1 probe, 1–6 MHz); i800 (Cannon Medical systems Corporation, Japan; i8CX1 probe, 1-8MHz); and Resona 7T (Mindray, China; SC6-1 U probe, 1-6MHz).

### 2.3 Histological and Immunohistochemistry

**Figure 2:**
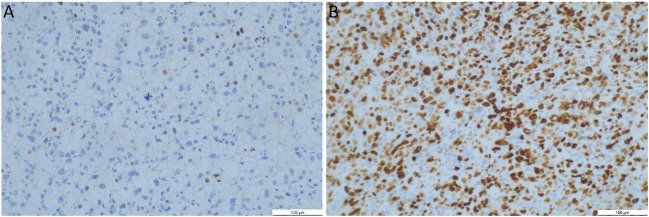
Representative immunohistochemistry Ki-67 staining patterns and dot plots assessing the percentage of Ki-67 staining cells (original magnification, 200x). **(A)** Low Ki-67 expression (7%); **(B)** High Ki-67 expression (80%). Brown-stained nuclei were considered positive Ki-67 expression.

The specimens were fixed in 3.7% neutral formaldehyde, paraffin-embedded, and cut into 4 mm thick sections. Ki-67 proliferation was detected using immunohistochemistry with the Ventana Benchmark Ultra automated staining system (Roche Ventana, Inc.). Tumor nuclei with brown-stained nuclei were considered positive for Ki-67 expression. The Ki-67 PI was calculated as the percentage of positive nuclei, and HCC lesions were classified into two groups based on their PI values: a high expression group (PI > 20%) and a low expression group (PI ≤ 20%) (24), as shown in **Figure 2**.

### 2.4 Tumor segmentation and feature extraction

**Figure 3:**
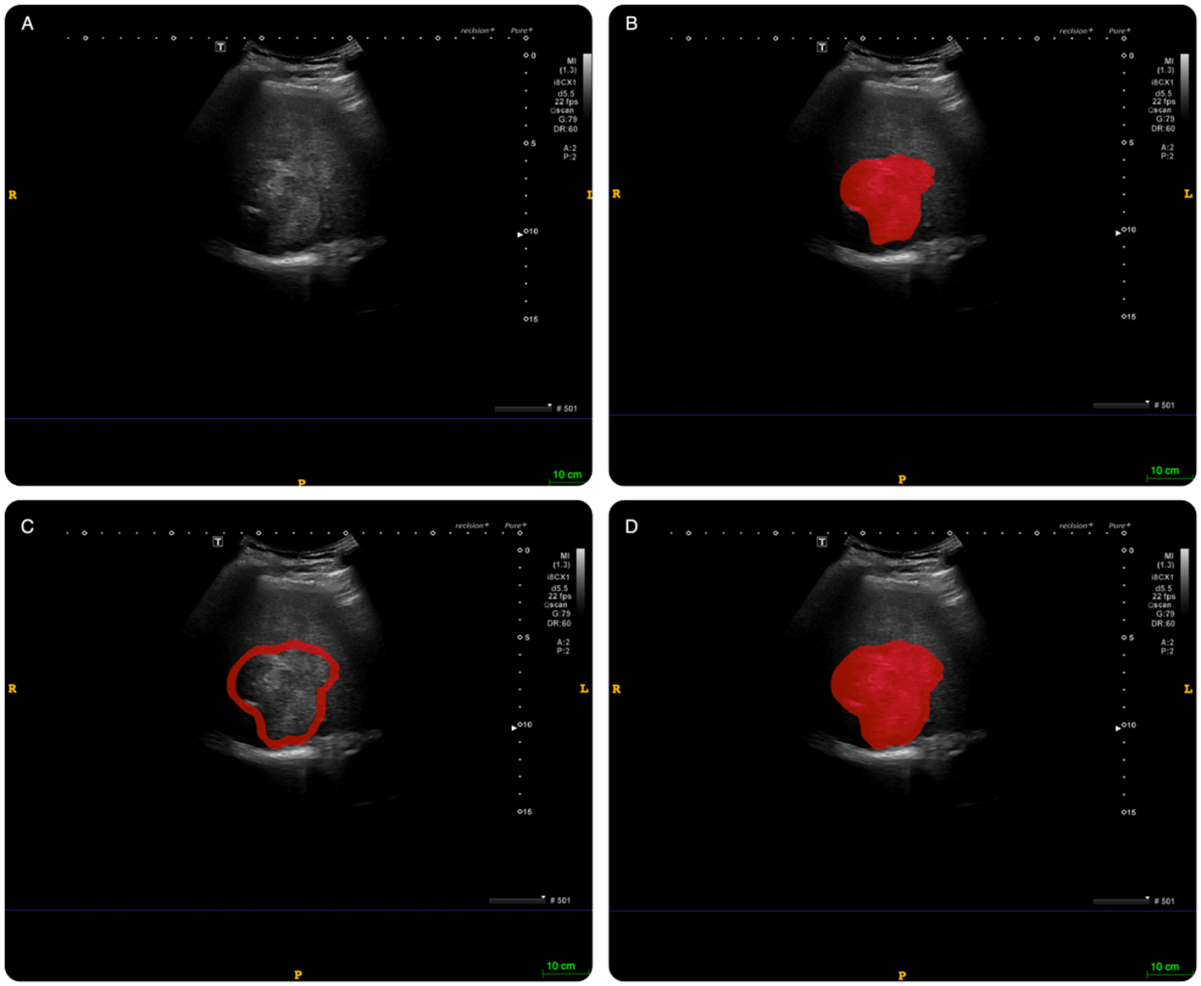
An example of delineating region of interest (ROI) on abdominal ultrasound imaging in ITK-SNAP software. **(A)**: original image; **(B)**: intratumoral ROI. **(C)**: peritumoral ROI; **(D)**: combined ROI (intratumoral tissue + peritumoral tissue).

The intratumoral region of interest (ROI) segmentation was manually delineated using ITK-SNAP software (Version 3.8.0, www.itksnap.org) along the tumors’ edge. The peritumoral tissue was defined as the tissue located at a distance of 2 cm from the tumor. If the peritumoral ROI extended beyond the liver tissue, the liver capsule was used as the boundary. This process was carried out independently by two experienced sonographers who were blinded to the patients’ clinical data, as illustrated in **Figure 3**, and was repeated one week later for consistency.

### 2.5 Feature extraction and dimension reduction

Before extracting features, we standardized all ultrasound images, which involved resampling the images to a uniform spatial resolution of 3 x 3 x 3 mm^3^, normalizing the intensity values to 32 grey levels using a normalization scale of 255, and eliminating any machine-specific artifacts or noise.

We utilized the Pyradiomics (open-source Python package) to extract high-order features from the original and filtered images, such as Wavelet, LoG, Square, SquareRoot, Logarithmic, Exponential, and Gradient filters, in addition to first- and second-order features from preoperative abdominal ultrasound images. Due to the wide range of magnitudes among the features, we applied standard Z-score scaling to normalize them.

To assess the reproducibility of the radiomics features, we computed inter- and intra-class coefficients (ICC). The inter-class coefficients were derived by comparing the features extracted by different sonographers, while the intra-class coefficients were obtained by comparing the features extracted at different times, one week apart. We only included features with ICCs above 0.8 in subsequent feature selection, indicating their reproducibility.

We utilized the variance method, t-test, and the least absolute shrinkage and selection operator (LASSO) to determine the optimal features.

### 2.6 Radiomics Model construction and evaluation

To identify the best-performing model, we constructed multiple models using various approaches. We evaluated each model using a receiver operating characteristic (ROC) curve and computed its area under the curve (AUC) value.

Parameter selection plays a crucial role in optimizing the performance of machine learning models. In this study, we employed a two-stage approach (RandomizedSearchCV—GridSearchCV) to select the best hyperparameters for multiple machine learning models, namely Support Vector Machine (SVM), Random Forest, K-Nearest Neighbors (KNN), Logistic Regression, and Artificial Neural Network (ANN) (**Supplement Material I**).

The model with the highest AUC in the validation group was selected as the top performer. The intratumoral and peritumoral models with the best performance were merged using Logistic regression. We employed the calibration curve to visually illustrate the agreement between pathologically confirmed Ki-67 status and the prediction of the merged model. Moreover, we used decision curve analysis (DCA) to assess the clinical utility of the models by estimating the benefits at various threshold probabilities.

### 2.7 Statistical analysis

All radiomics procedures and statistical analyses were conducted with Python software (Version 3.8.5). R language (Version 4.2.2, R Foundation for Statistical Computing, Vienna, Austria) was used for waterfall plots, calibration curves, and DCA. Continuous variables were compared using t-test or Mann-Whitney U test, and categorical variables were compared with chi-square test. P values less than 0.05 were regarded as statistically significant.

## 3 Results

### 3.1 Characteristics of the study population

A total of 118 patients were ultimately included in this study, with 64 being confirmed to have high Ki-67 expression and 54 with low Ki-67 expression. These patients were randomly divided into training (n = 82) and validation (n = 36) groups. **Table 1** presents the clinical characteristics of all patients, and there were no significant differences between the high and low Ki-67 expression groups in terms of all clinical characteristics (p > 0.05). Moreover, we compared the clinical characteristics of the high and low Ki-67 expression groups in the training and validation sets. We found a significant difference in the mean age of the high Ki-67 expression group in the validation set compared to the low Ki-67 expression group (62.16 ± 6.86 vs 70.35 ± 10.89, p = 0.012). However, there were no significant differences in other clinical characteristics (p > 0.05) (**Table 2**).

**Table 2:** Comparison of clinical characteristics between the high Ki-67 expression group and low Ki-67 expression group in training and validation groups.

Variables	Training Group		P	Validation Group		P
	High Ki-67	Low Ki-67		High Ki-67	Low Ki-67	
Age(year)	64.61 ± 12.19	64.58 ± 10.49	0.992	62.16 ± 6.86	70.35 ± 10.89	0.012*
Sex			0.989			0.199
Male	37	29		12	14	
Female	9	7		7	3	
HBsAg			0.544			0.090
Positive	29	25		16	10	
Negative	17	11		3	7	
AFP (mg/mL)	2739.34 ± 12637.85	1077.99 ± 5666.61	0.471	1861.95 ± 6613.78	351.69 ± 961.05	0.371
Alb (g/L)	38.5 ± 4.39	38.91 ± 4.5	0.685	38.88 ± 4.83	38.8 ± 3.03	0.955
ALT(IU/L)	37.83 ± 26.06	47.1 ± 41.67	0.227	39.25 ± 56.07	26.62 ± 17.36	0.393
AST(IU/L)	40.86 ± 24.24	49.87 ± 44.26	0.25	46.75 ± 77.93	38.31 ± 21.91	0.678
TBIL (µmol/L)	18.18 ± 22.51	17.64 ± 9.48	0.893	17.41 ± 12.43	13.16 ± 5.9	0.22
DBIL (µmol/L)	6.58 ± 13.31	6.35 ± 4.79	0.924	5.22 ± 4.0	5.62 ± 5.63	0.81
PT (s)	12.97 ± 1.14	13.33 ± 1.33	0.196	13.44 ± 1.9	12.84 ± 1.29	0.289
INR						
Tumor Size (cm)	1.04 ± 0.1	1.06 ± 0.11	0.306	1.07 ± 0.2	1.05 ± 0.09	0.68
Cirrhosis			0.696			0.158
Absent	21	18		9	12	
Present	25	18		10	5	
Multifocality			0.362			0.797
Absent	36	31		15	14	
Present	10	5		4	4	

AFP, alpha fetoprotein; ALB, albumin level; ALT, alanine aminotransferase; AST, aspartate aminotransferase; TBIL, total bilirubin; DBIL, directed bilirubin; PT, prothrombin time; INR, international normalized ratio.

### 3.2 Feature selection

We initially extracted 1595 features from both the original and filtered images, which were further refined by excluding 6 features in the intratumoral model, 19 features in the peritumoral model, and 22 features in combined model#2 based on their intra- and inter-class coefficients. To reduce dimensionality, we removed low variance radiomics features and those that were highly correlated (with an absolute Pearson correlation greater than 0.8) with any other feature. Further refinement was performed using T-test and LASSO regression, resulting in 7 radiomics features in the intratumoral model, 8 radiomics features in the peritumoral model, and 5 radiomics features in combined model#2 (**Table 3**). The detailed LASSO results of the intratumoral model, peritumoral model, and combined model#2 can be found in **Supplementary Figure 1**-**3**.

**Table 3:** The finally selected radiomics features and their coefficient values.

Model	Filter	Feature class	Feature	Coefficient
**Intratumoral model**	wavelet-LHH	glrlm	HighGrayLevelRunEmphasis	0.06926744
	wavelet-LHH	glrlm	LowGrayLevelRunEmphasis	-3.22125E-15
	wavelet-HLH	glcm	SumEntropy	0.07984176
	wavelet-HHH	firstorder	Skewness	0.02174849
	wavelet-LLL	glszm	SmallAreaHighGrayLevelEmphasis	0.06275222
	square	glszm	LowGrayLevelZoneEmphasis	-0.02366083
	exponential	glszm	GrayLevelVariance	-0.03274517
**Peritumoral model**	wavelet-HLL	glszm	SmallAreaHighGrayLevelEmphasis	-0.001741
	wavelet-HLL	ngtdm	Strength	-0.059366
	wavelet-HHH	glszm	SizeZoneNonUniformityNormalized	0.004511
	wavelet-LLL	glszm	SmallAreaHighGrayLevelEmphasis	0.049172
	exponential	glcm	Idn	-0.05651
	exponential	glszm	ZoneVariance	0.0323
	logarithm	glrlm	ShortRunLowGrayLevelEmphasis	-0.134367
	gradient	ngtdm	Strength	-0.060406
**Combined model#2**	wavelet-LHL	firstorder	Mean	-0.05218
	wavelet-HLH	firstorder	Mean	0.047801
	wavelet-LLL	glszm	SmallAreaHighGrayLevelEmphasis	0.113069
	exponential	glcm	Idn	-0.036239
	gradient	ngtdm	Strength	-0.081881

### 3.3 Intratumoral and peritumoral model

We utilized various modeling techniques, including Support Vector Machine (SVM), Random Forest (RF), K Nearest Neighbor (KNN), Logistic Regression (LR), and Artificial Neural Network (ANN), to identify the best modeling method.

In the intratumoral model, the SVM algorithm had an AUC of 0.796 (0.649-0.942), the RF algorithm had an AUC of 0.786 (0.637-0.936), the KNN algorithm had an AUC of 0.772 (0.619-0.926), the LR algorithm had an AUC of 0.777 (0.625-0.93), and the ANN algorithm had an AUC of 0.765 (0.609-0.921). The SVM classifier exhibited the highest performance in predicting Ki-67 expression in the intratumoral model.

**Figure 4:**
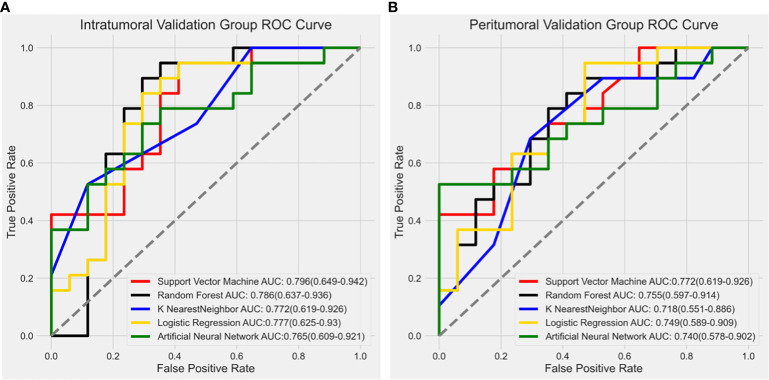
Receiver operating characteristic (ROC) curve results from different modeling methods in the intratumoral model and peritumoral model. The SVM algorithm showed the highest diagnostic performance with an AUC value of 0.796 (0.649-0.942) in the intratumoral model **(A)** and 0.772 (0.619-0.926) in the peritumoral model **(B)**.

Similarly, the SVM algorithm was the most effective in the peritumoral model with an AUC of 0.772 (0.619-0.926), while the RF algorithm had an AUC of 0.755 (0.597-0.914), the KNN algorithm had an AUC of 0.718 (0.551-0.886), the LR algorithm had an AUC of 0.749 (0.589-0.909), and the ANN algorithm had an AUC of 0.740 (0.578-0.902). The ROC curves for the validation groups in the intratumoral and peritumoral models were displayed in **Figure 4**.

**Figure 5:**
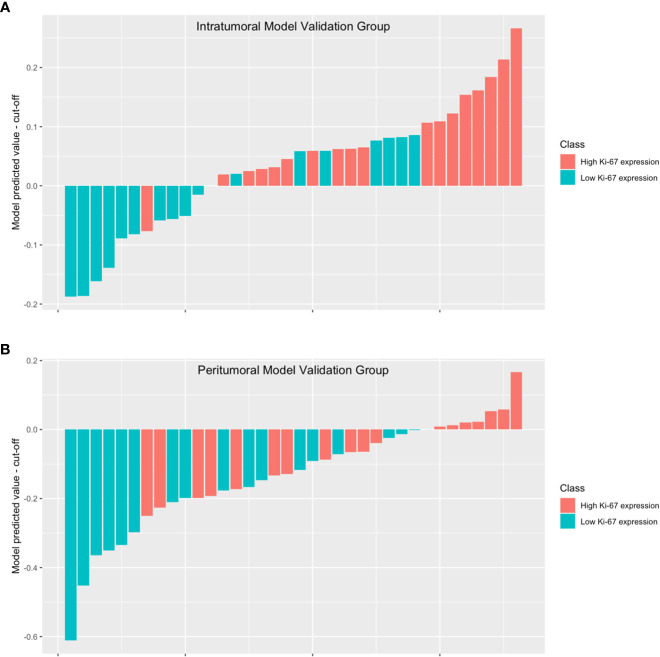
The waterfall plot displays the model’s performance in the intratumoral model validation group **(A)** and the peritumoral model validation group **(B)**. The height of each bar in the chart represented the model predicted value minus the cut-off. The bars above the y=0 line indicate that the model predicts high Ki-67 expression, while the bars below the y=0 line indicate that the model predicts low Ki-67 expression.

To better illustrate the performance of the model, we plotted a waterfall plot (**Figure 5**). The height of each bar in the chart represented the model predicted value minus the cut-off. The bars above the y=0 line indicate that the model predicts high Ki-67 expression, while the bars below the y=0 line indicate that the model predicts low Ki-67 expression. The results demonstrated the reliability of the intratumoral model and the peritumoral for evaluating Ki-67 expression levels in patients with HCC.

### 3.4 Combined model

The combined model was built in two different ways: Combined model#1, using logistic analysis to combine the most effective intratumoral and peritumoral models; Combined model#2, by drawing the ROI, in which both the tumor and the tumor margin tissues were included.

**Figure 6:**
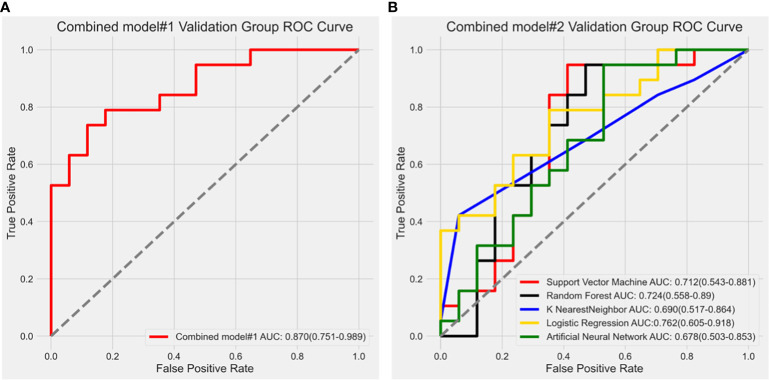
**(A)** Receiver operating characteristic curve analysis of combined model#1; **(B)** Receiver operating characteristic (ROC) curve results of different modeling methods in combined model#2. The LR algorithm showed the highest diagnostic performance with an AUC value of 0.762 (0.605-0.918).

**Figure 7:**
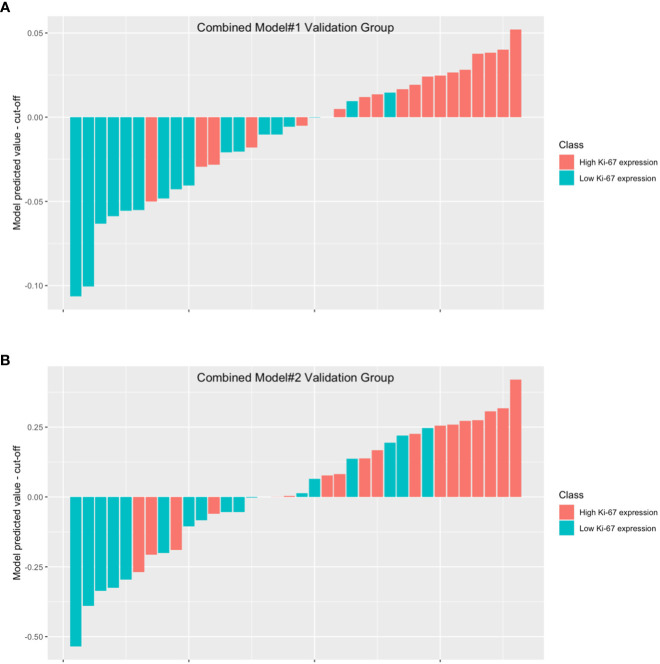
The waterfall plot displays the model’s performance in combined model#1 validation group **(A)** and combined model#2 validation group **(B)**. The height of each bar in the chart represented the model predicted value minus the cut-off. The bars above the y=0 line indicate that the model predicts high Ki-67 expression, while the bars below the y=0 line indicate that the model predicts low Ki-67 expression.

In combined model#1, we adopted logistic regression to coalesce the outcome of the intratumoral model and the peritumoral model. The AUC of combined model#1 was 0.870 (0.751-0.989). According to the Delong test, we observed that combined model#1 demonstrated a significantly higher diagnostic efficacy in comparison to the peritumoral model (p < 0.05), yet not surpassing the intratumoral model (p = 0.269). In combined Model#2, akin to the intratumoral and peritumoral models, various modeling approaches were employed in the model building. The maximum AUC of combined Model#2 was 0.762 (0.605-0.918) with the application of a LR classifier (**Figure 6**). Both combined model#1 and combined model#2 could predict Ki-67 expression accurately (**Figure 7**).

No noteworthy disparity existed in the diagnostic prowess of combined model#2 in comparison to that of the intratumoral model (p = 0.734) or the peritumoral model (p = 0.596). Nevertheless, the diagnostic capability of combined model#2 was inferior to that of combined model#1 (p < 0.05).

**Figure 8:**
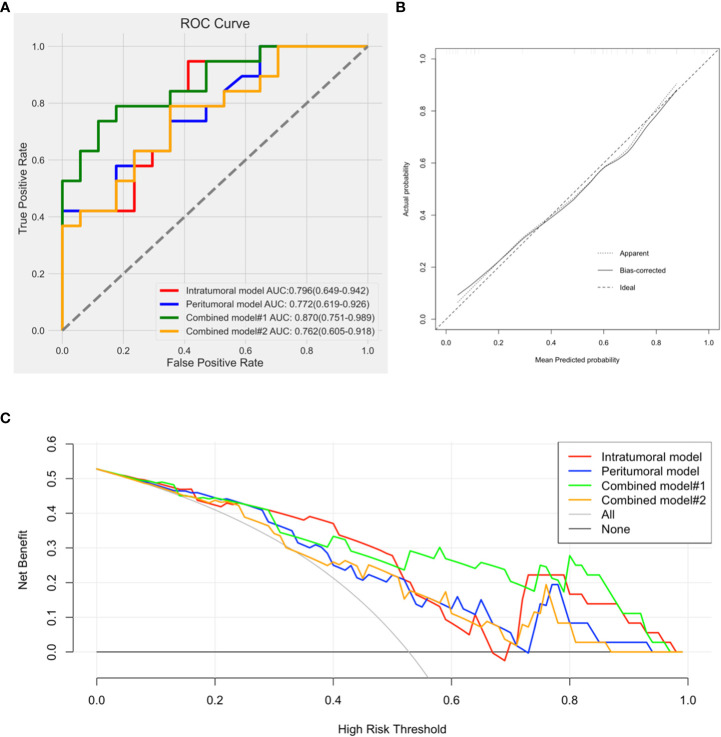
**(A)**. Comparison of receiver operating characteristic curves for differentiation of the four models; **(B)**. Calibration curve of combined model#1 presented a good agreement between the predicted and pathologically confirmed Ki-67 status with dotted line (actual calibration) closed to dashed line (perfect calibration); **(C)**. Decision curves for the intratumoral model, peritumoral model, combined model#1, and combined model#2.

Combined model#1 demonstrated the most noteworthy AUC in the four models. Calibration graphs of combined model#1 and Hosmer-Lemeshow test exposed a satisfactory concurrence between the anticipated and pathologically affirmed Ki-67 status (P = 0.663). Using DCA, the performances of all models were evaluated. Each model showed a higher area under the decision curve than the “treat all” (solid gray line) or “treat none” (dotted gray line) approaches. Out of the four prediction models, combined model#1 had the greatest area under the decision curve and exhibited the most benefit over a wide range of threshold probabilities (**Figure 8**).

We observed combined model#1 had the largest AUC, accuracy, and F1-score in the four models (**Table 4**).

**Table 4:** The performance of the intratumoral model, peritumoral model, combined model#1, and combined model#2 in predicting Ki-67 expression.

	Intratumoral model	Peritumoral model	Combined model#1	Combined model#2
tp	18	8	14	15
fp	7	0	2	6
fn	1	11	5	4
tn	10	17	15	11
AUC	0.796	0.772	0.87	0.762
Sensitivity (%)	94.74%	42.11%	73.68%	78.95%
Specificity (%)	58.82%	100.00%	88.24%	64.71%
Precision (%)	72.00%	100%	87.50%	71.43%
Recall (%)	94.74%	42.11%	73.68%	78.95%
Accuracy (%)	78%	69.44%	80.56%	72.22%
F1-score	0.8182	0.5926	0.8	0.75

tp, true positive; fp, false positive; fn, false negative; tn, true negative; AUC, area under the curve.

## 4 Discussion

We evaluated the performance of an ultrasound radiomics model for preoperative prediction of Ki-67 expression in HCC patients. The study showed that the model, based on both intratumoral and peritumoral tissues, exhibited excellent performance and generalization ability in predicting Ki-67 expression. As far as we know, this is the first study to assess the reliability of such an ultrasound radiomics model that combines both intratumoral and peritumoral tissues for predicting Ki-67 expression.

In our study, both the radiomics models based on intratumoral and peritumoral tissues demonstrated accurate prediction of Ki-67 expression. The intratumoral model had an AUC of 0.796(0.649-0.942), while the peritumoral model had an AUC of 0.772(0.619-0.926). Furthermore, the Delong test revealed no significant difference in diagnostic performance between the two models, suggesting that peritumoral tissues also provide valuable information for predicting Ki-67 expression.

Our study combined both intra-tumor and peri-tumor tissues in the analysis, providing a more comprehensive representation of the tissue microenvironment surrounding the tumor. Combined model#1 showed better diagnostic performance than the peritumoral model, but not the intratumoral model. This result demonstrated the capability of the intratumoral model. Although no significant difference was found between the intratumoral and combined model#1, incorporating the peritumoral model contributed to a larger AUC, accuracy, F1-score, and a better DCA result. Compared to other radiomics models that only focus on intratumoral tissue, our study may provide improved accuracy and robustness in predicting Ki-67 expression in HCC. The additional information from the peritumoral tissue can help to better capture the tumor biology and heterogeneity as supplemental data in combined model#1.

We combined intratumoral and peritumoral tissues in two ways: using logistic regression to combine the models, or including both regions in the ROI. The combined model using logistic regression achieved better diagnostic performance, likely because it takes into account the interplay between the intratumoral and peritumoral features in a more sophisticated way. Logistic regression is a statistical model that can be used to predict a binary outcome based on a set of input features (25). In our study, the outcome was the expression of Ki-67 in HCC, and the input features were the radiomics features from both the intratumoral and peritumoral regions. By using logistic regression to combine the radiomics models, we can take into account the interplay between the intratumoral and peritumoral features in a more nuanced way. The logistic regression model can learn the relationship between the features, and weigh each feature according to its contribution to the prediction. This can help to identify the most important features and improve the overall accuracy of the model. In contrast, simply including both the intratumoral and peritumoral regions in the ROI may not fully capture the interplay between the features. This approach may also result in larger ROIs, which can increase the noise and variability in the features, and may not effectively capture the most important features for the prediction (26, 27). We think the use of logistic regression to combine the radiomics models was a more sophisticated approach that can take into account the relationship between the features in a more effective way, and can help to improve the accuracy of the prediction of Ki-67 expression in HCC.

Radiomics features can be grouped into first-order, second-order, and high-order ones. First-order, or histogram features, refer to gray-level values of single voxels, not considering spatial correlations. Second-order, typically called texture analysis, depict spatial relationships between voxels with similar gray levels within lesions (28), usually GLCM and GLRLM being used to indicate tumor heterogeneity and complexity (29, 30). High-order features are derived from filters applied to images, such as Wavelet, LoG, Square, SquareRoot, Exponential, Logarithm, and Gradient (31), gathering more hidden information from images (32). Most radiomics models were based on low-order features (first-order or second-order), with little evidence of whether whole-order radiomics features (first-order, second-order, and high-order) can predict Ki-67 expression in HCC patients. We obtained eight radiomics features, five of them being wavelet features, the result suggesting wavelet is essential for predicting pathological results, as in previous studies (33, 34). Wavelet filter divides images into sub-images of different frequency components, allowing to explore spatial heterogeneity within ROIs at multiple scales (34). Wavelets are mathematical functions that can be used to analyze signals and images in a multi-scale manner, allowing for the extraction of features at different levels of detail. In the context of HCC evaluation, wavelet features may be useful in capturing the complex patterns of Ki-67 expression that are associated with the cellular proliferation and aggressiveness of the tumor. Additionally, wavelets have been successfully used in other imaging-based studies to extract features that are indicative of specific biological processes (35). The fact that wavelet features were found to be the most predictive in our study suggested that they are capturing relevant information about Ki-67 expression in HCC and that they may be particularly well-suited for this type of analysis. In our opinion, the use of wavelet features in predicting Ki-67 expression in HCC highlights the potential of multi-scale analysis for radiomics studies and provides a promising direction for future research in this area.

There is ongoing research to identify clinical parameters that can accurately predict Ki-67 expression preoperatively in patients with HCC. Currently, there is no established clinical parameter or combination of parameters that can reliably predict Ki-67 expression in HCC. However, several factors have been associated with increased Ki-67 expression, including larger tumor size, higher serum alpha-fetoprotein levels, and the presence of vascular invasion (15, 24, 36). Other factors, such as age, underlying liver disease, and liver function, may also play a role in determining Ki-67 expression in HCC (37, 38). In our research, only age was found to be significantly different between the high Ki-67 expression group and the low Ki-67 expression group in the validation set. And there was no significant difference in other clinical parameters between low and high ki-67 expression groups. We think the relationship between clinical parameters and Ki-67 expression in HCC is complex and predicting Ki-67 expression only by clinical parameters is difficult.

Our research has demonstrated that ultrasound based radiomics analysis of intratumoral and peritumoral tissues can provide valuable pathological information about HCC. We hope that our study can offer insights into challenging issues, such as the monitoring strategy for early or very early stages of liver cancer, in which the exact value of ultrasound is subject to certain controversies. Park HJ et al. (39) thought that while ultrasound holds some value in the surveillance of HCC, its sensitivity is relatively low, particularly in the early and very early stages of liver cancer. Therefore, it is recommended to complement ultrasound with other imaging techniques such as CT and MRI. However, both the Japanese guidelines and the American Association for the Study of Liver Diseases guidelines endorse ultrasound surveillance for early detection of HCC in patients with cirrhosis or chronic hepatitis B. Furthermore, the ultrasound contrast agent Sonazoid can provide insights into the blood supply features of HCC, assisting in determining the need for treatment in low-vascularity nodules smaller than 1cm (21). A meta-analysis suggested combining ultrasound with AFP significantly increases early HCC detection, making it a preferred surveillance strategy for cirrhosis patients until better options are available (40). We hope radiomics may provide valuable insights to help ultrasound overcome these limitations (41).

There were some limitations in our study: First, this was a single-center study, which may not represent the population of interest as a whole. Second, the sample of our study was small. The small sample size may limit the ability to control for potential confounding factors, such as age, sex, and comorbidities, which may affect outcomes. Third, this was a retrospective study, which may introduce potential selective bias. Fourth, the influence of different ultrasound devices was not analyzed in our study, which may affect the reproducibility and generalizability of the radiomics model. Fifth, it should be noted that all patients included in our study were surgical cases, which might introduce a certain degree of selection bias as individuals with advanced stages who were not eligible for surgical treatment were not included in the study. This limitation may impact the generalizability of the findings to the broader patient population with the condition under investigation. Finally, we did not select the entire tumor as the ROI but chose the section where the tumor had the maximum diameter. This may lead to the loss of partial information, and the obtained radiomic features may not represent the entire tumor.

## 5 Conclusion

We provided an ultrasound radiomics model combining intratumoral and peritumoral tissues which could accurately predict Ki-67 expression in patients with HCC patients. By combining both intratumoral and peritumoral information, our model may provide a more comprehensive picture of the disease and may help to improve patient prognosis and treatment planning. According to our view, the use of both intratumoral and peritumoral tissue in the radiomics model was a unique and valuable approach that can provide new insights into the biology of HCC and help to improve patient care.

## Data availability statement

The raw data supporting the conclusions of this article will be made available by the authors, without undue reservation.

## Ethics statement

The studies involving humans were approved by Ethics Committee of Shaoxing people’s Hospital. The studies were conducted in accordance with the local legislation and institutional requirements. The participants provided their written informed consent to participate in this study.

## Author contributions

HQ: Methodology, Software, Validation, Writing - Original Draft, Funding; ZS: Conceptualization, Methodology, Visualization, Supervision; DZ: Investigation, Data Curation; YH: Data Curation, Writing - Review & Editing, Formal analysis. All authors contributed to the article and approved the submitted version
