# Supplementary figures and images for: Intratumoral and peritumoral radiomics model based on abdominal ultrasound for predicting Ki-67 expression in patients with hepatocellular cancer
Source: Front Oncol. 2023 Aug 24;13:1209111. doi: 10.3389/fonc.2023.1209111 (PMC10498123; doi:10.3389/fonc.2023.1209111)

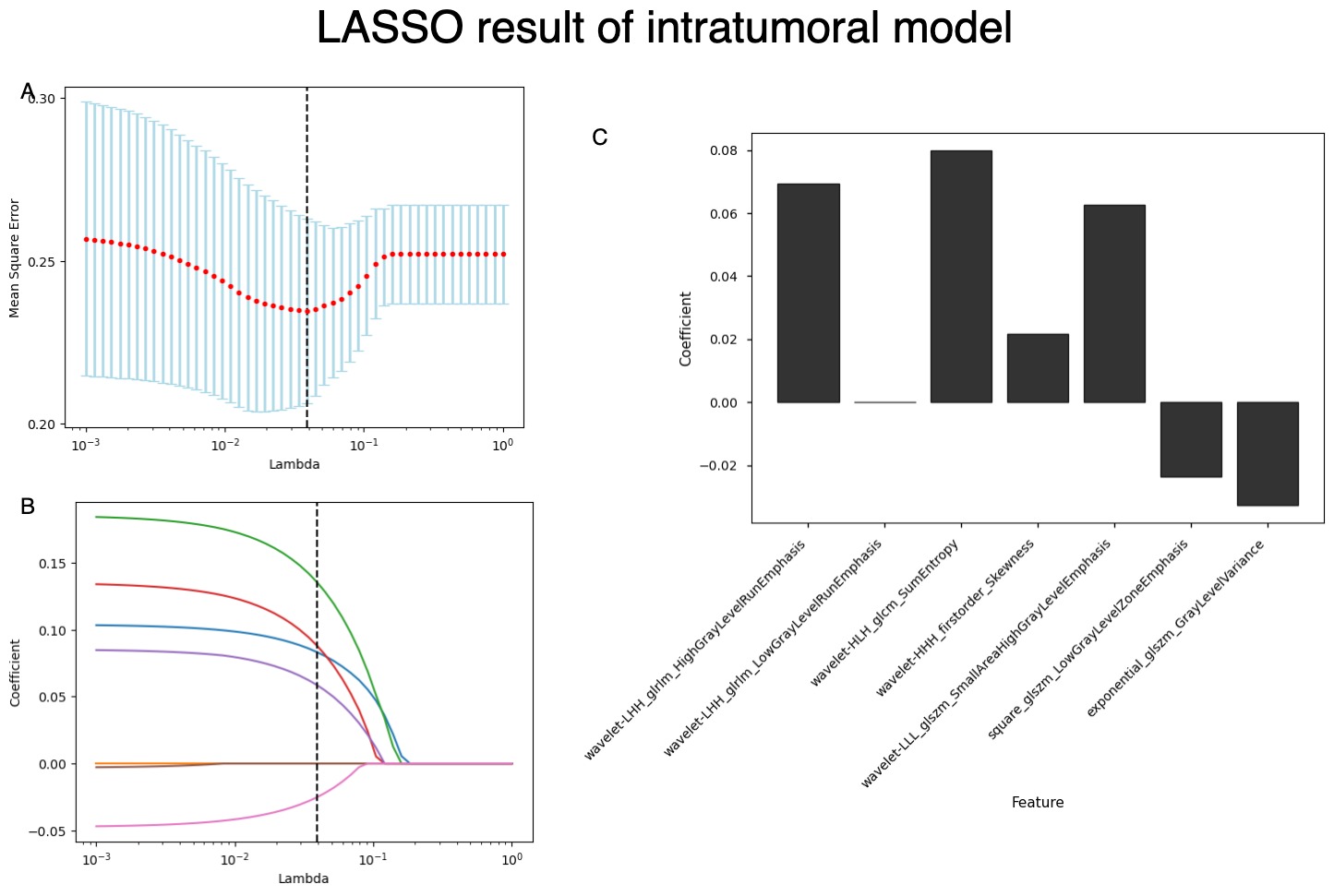

Supplement: Supplementary file 1 [file Image_1.jpeg]

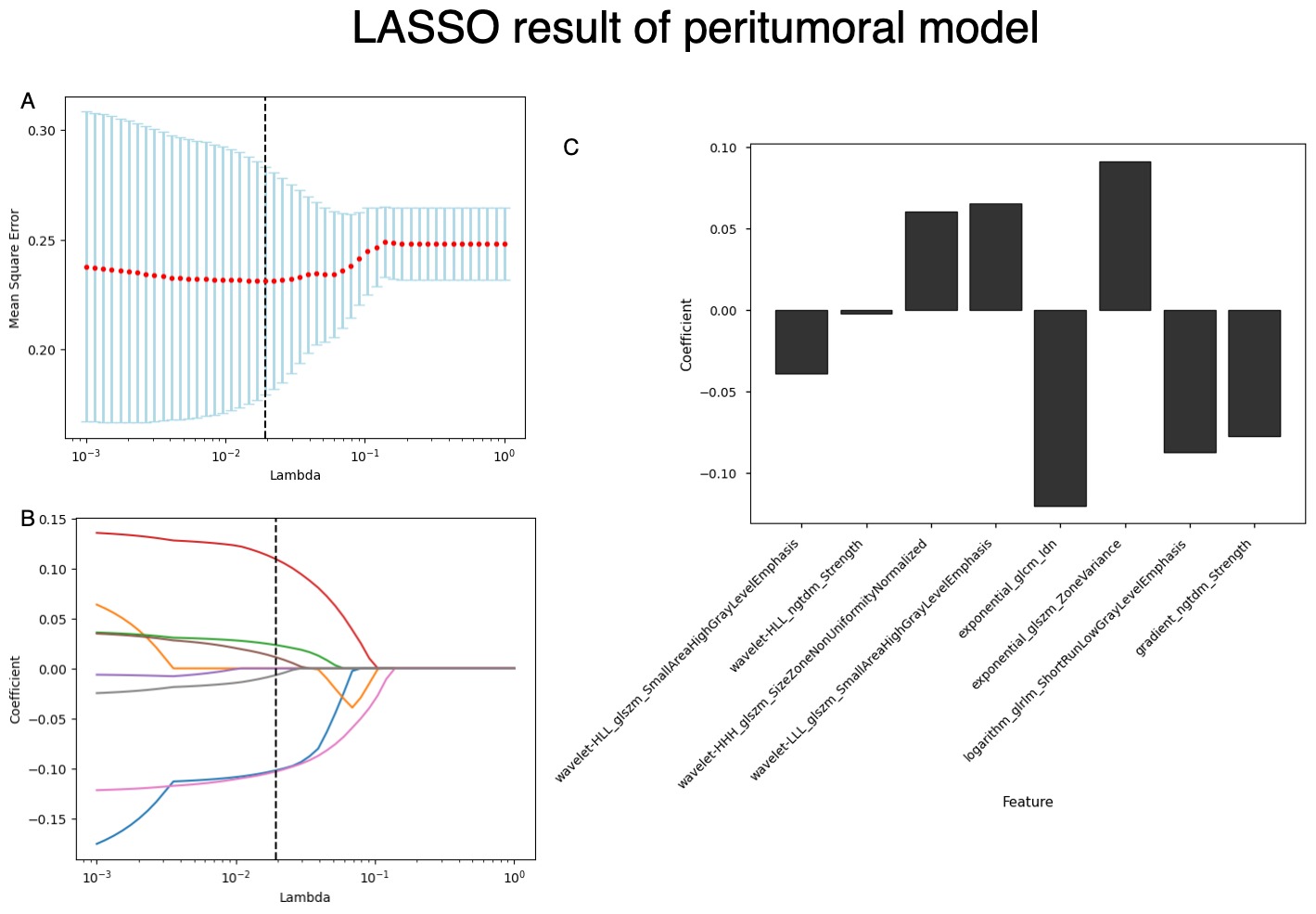

Supplement: Supplementary file 2 [file Image_2.jpeg]

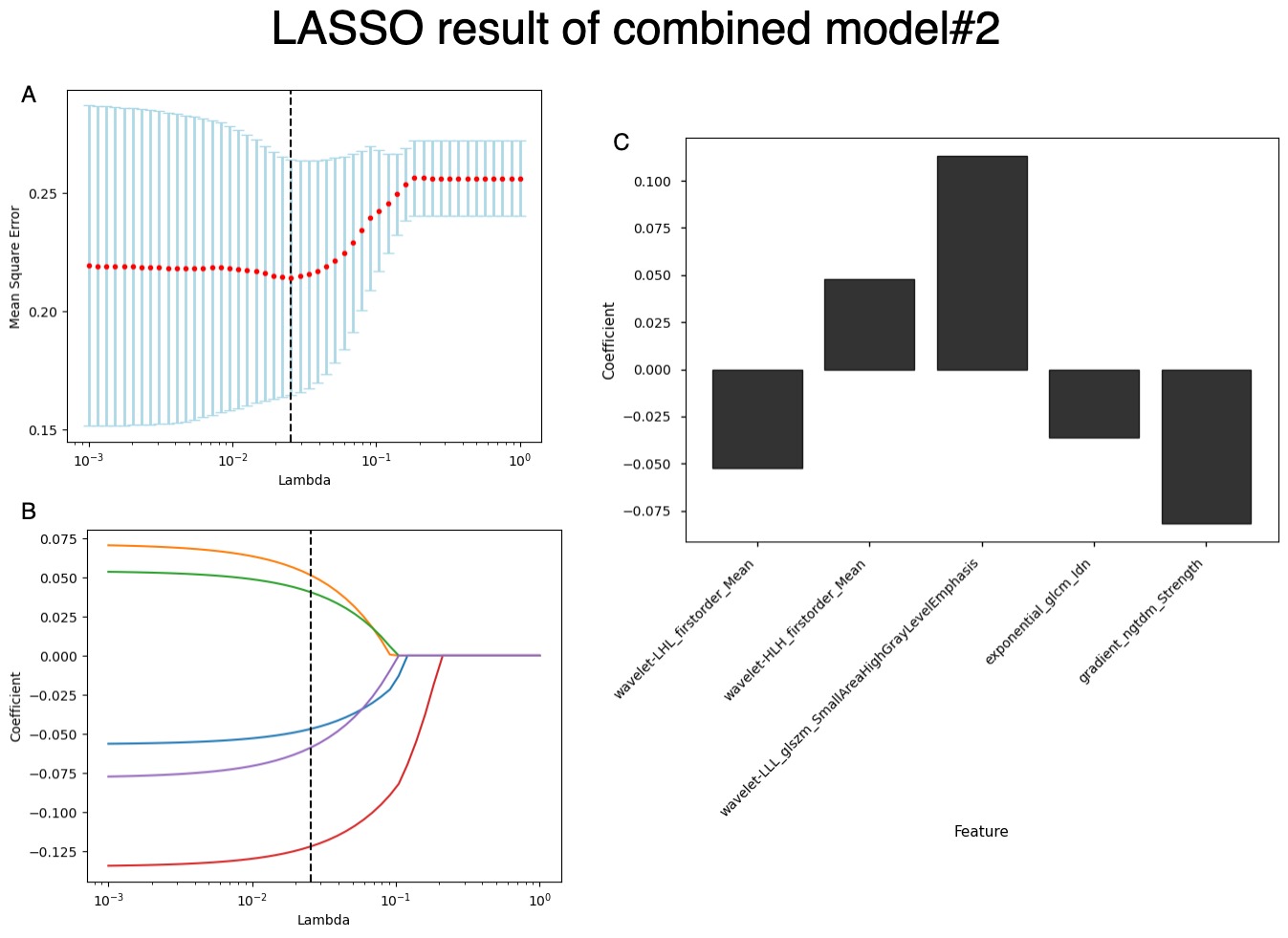

Supplement: Supplementary file 3 [file Image_3.jpeg]
