# Supplementary material for: Intratumoral and peritumoral radiomics model based on abdominal ultrasound for predicting Ki-67 expression in patients with hepatocellular cancer
Source: Front Oncol. 2023 Aug 24;13:1209111. doi: 10.3389/fonc.2023.1209111 (PMC10498123; doi:10.3389/fonc.2023.1209111)
Supplement: Supplementary file 4 [file Table_1.docx]

TABLE S1

Comparison of clinical characteristics between the training group and testing groups

| **Variables** | **Training Group** | **Testing Group** | **P** |
| --- | --- | --- | --- |
| **Age(year)** | 64.6 ± 11.48 | 66.03 ± 9.88 | 0.521 |
| **Sex** |  |  | 0.319 |
| Male | 66 | 26 |  |
| Female | 16 | 10 |  |
| **HBsAg** |  |  | 0.495 |
| Positive | 54 | 26 |  |
| Negative | 28 | 10 |  |
| **AFP (mg/mL)** | 2009.96 ± 10216.33 | 1148.77 ± 4908.23 | 0.634 |
| **Alb (g/L)** | 38.68 ± 4.45 | 38.84 ± 4.08 | 0.851 |
| **ALT(IU/L)** | 41.9 ± 34.12 | 33.29 ± 42.91 | 0.251 |
| **AST(IU/L)** | 44.82 ± 34.78 | 42.76 ± 58.73 | 0.815 |
| **TBIL (µmol/L)** | 17.94 ± 17.99 | 15.4 ± 10.12 | 0.433 |
| **DBIL (µmol/L)** | 6.48 ± 10.46 | 5.41 ± 4.84 | 0.561 |
| **PT (s)** | 13.13 ± 1.24 | 13.16 ± 1.67 | 0.915 |
| **INR** | 1.05 ± 0.11 | 1.06 ± 0.16 | 0.642 |
| **Tumor Size(cm)** | 4.9 ± 2.83 | 4.95 ± 2.75 | 0.924 |
| **Cirrhosis** |  |  | 0.281 |
| Absent | 39 | 21 |  |
| Present | 43 | 15 |  |
| **Multifocality** |  |  | 0.882 |
| Absent | 67 | 29 |  |
| Present | 15 | 7 |  |

AFP, alpha fetoprotein; ALB, albumin level; ALT, alanine aminotransferase; AST, aspartate aminotransferase; TBIL, total bilirubin; DBIL, directed bilirubin; PT, prothrombin time; INR, international normalized ratio
