# Supplementary material for: Intratumoral and peritumoral radiomics model based on abdominal ultrasound for predicting Ki-67 expression in patients with hepatocellular cancer
Source: Front Oncol. 2023 Aug 24;13:1209111. doi: 10.3389/fonc.2023.1209111 (PMC10498123; doi:10.3389/fonc.2023.1209111)
Supplement: Supplementary file 5 [file Table_2.docx]

**Best parameters in the intratumoral model**

Best parameters for SVM from RandomizedSearch: {'kernel': 'linear', 'gamma': 376.49358067924715, 'C': 0.40370172585965536}

Best parameters for SVM from GridSearch: {'C': 0.11245398776451322, 'gamma': 37.649358067924716, 'kernel': 'linear'}

Best parameters for RandomForest from RandomizedSearch: {'n_estimators': 300, 'min_samples_split': 2, 'min_samples_leaf': 1, 'max_depth': 20, 'bootstrap': True}

Best parameters for RandomForest from GridSearch: {'bootstrap': True, 'max_depth': 20, 'min_samples_leaf': 1, 'min_samples_split': 2, 'n_estimators': 300}

Best parameters for KNN from RandomizedSearch: {'weights': 'distance', 'n_neighbors': 9, 'algorithm': 'auto'}

Best parameters for KNN from GridSearch: {'algorithm': 'auto', 'n_neighbors': 9, 'weights': 'distance'}

Best parameters for LogisticRegression from RandomizedSearch: {'solver': 'liblinear', 'penalty': 'l2', 'C': 0.004037017258596553}

Best parameters for LogisticRegression from GridSearch: {'C': 0.0004037017258596554, 'penalty': 'l2', 'solver': 'liblinear'}

Best parameters for ANN from RandomizedSearch: {'hidden_layer_sizes': (100,), 'alpha': 0.029836472402833405, 'activation': 'tanh'}

Best parameters for ANN from GridSearch: {'activation': 'tanh', 'alpha': 0.029836472402833405, 'hidden_layer_sizes': (100,)}

**Best parameters in the peritumoral model**

Best parameters for SVM from RandomizedSearch: {'kernel': 'linear', 'gamma': 0.01873817422860384, 'C': 0.049770235643321115}

Best parameters for SVM from GridSearch: {'C': 0.049770235643321115, 'gamma': 0.0018738174228603841, 'kernel': 'linear'}

Best parameters for RandomForest from RandomizedSearch: {'n_estimators': 50, 'min_samples_split': 10, 'min_samples_leaf': 2, 'max_depth': 10, 'bootstrap': True}

Best parameters for RandomForest from GridSearch: {'bootstrap': True, 'max_depth': 10, 'min_samples_leaf': 2, 'min_samples_split': 10, 'n_estimators': 50}

Best parameters for KNN from RandomizedSearch: {'weights': 'uniform', 'n_neighbors': 3, 'algorithm': 'brute'}

Best parameters for KNN from GridSearch: {'algorithm': 'brute', 'n_neighbors': 3, 'weights': 'uniform'}

Best parameters for LogisticRegression from RandomizedSearch: {'solver': 'liblinear', 'penalty': 'l2', 'C': 0.024770763559917114}

Best parameters for LogisticRegression from GridSearch: {'C': 0.014862458135950269, 'penalty': 'l2', 'solver': 'liblinear'}

Best parameters for MLPClassifier from RandomizedSearch: {'hidden_layer_sizes': (50,), 'alpha': 0.014174741629268062, 'activation': 'relu'}

Best parameters for MLPClassifier from GridSearch: {'activation': 'relu', 'alpha': 0.014174741629268062, 'hidden_layer_sizes': (50,)}

**Best parameters in combined model#2**

Best parameters for SVM from RandomizedSearch: {'kernel': 'linear', 'gamma': 0.8111308307896873, 'C': 0.049770235643321115}

Best parameters for SVM from GridSearch: {'C': 0.06817225663769536, 'gamma': 0.24499461827933416, 'kernel': 'linear'}

Best parameters for RandomForest from RandomizedSearch: {'n_estimators': 100, 'min_samples_split': 2, 'min_samples_leaf': 1, 'max_depth': 10, 'bootstrap': False}

Best parameters for RandomForest from GridSearch: {'bootstrap': False, 'max_depth': 10, 'min_samples_leaf': 1, 'min_samples_split': 2, 'n_estimators': 100}

Best parameters for KNN from RandomizedSearch: {'weights': 'uniform', 'n_neighbors': 3, 'algorithm': 'brute'}

Best parameters for KNN from GridSearch: {'algorithm': 'brute', 'n_neighbors': 3, 'weights': 'uniform'}

Best parameters for LogisticRegression from RandomizedSearch: {'solver': 'liblinear', 'penalty': 'l1', 'C': 497.7023564332114}

Best parameters for LogisticRegression from GridSearch: {'C': 109.01576664959921, 'penalty': 'l1', 'solver': 'liblinear'}

Best parameters for ANN from RandomizedSearch: {'hidden_layer_sizes': (50, 50), 'alpha': 0.00037649358067924713, 'activation': 'tanh'}

Best parameters for ANN from GridSearch: {'activation': 'tanh', 'alpha': 0.00037649358067924713, 'hidden_layer_sizes': (50, 50)}
